# 

**Published:** 1891-05-30

**Authors:** 


PRICE ONE PENNY.
No. 244, Vol. X.— May 30th, 1891.
[Registered at the General Post Office as a Newspaper for transmission in the United Kingdom and Abroad.]

WITH EXTRA NURSING SUPPLEMENT.

Per Annum, 6s. 6d.; post free.

Monthly Parts, 6d.; post free 8d.

Ditto per Annum, 8s., post free.

### THE HOSPITAL

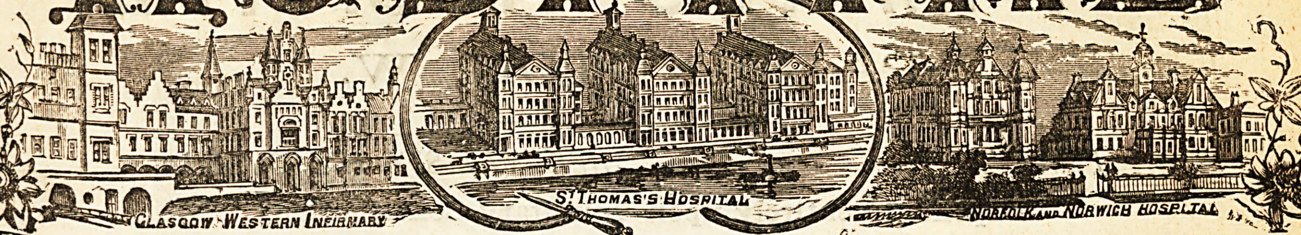

A WEEKLY JOURNAL OF

Science, Medicine, Nursing and Philanthropy.

### THE HOSPITAL SUNDAY NUMBER.

SATURDAY, MAY 30th, 1891.

**Under Which Flag?** - 97

**Passing Topics.**—Hospital Sunday - 98

**Metropolitan Hospital Sunday Fund.**—Facts for Preachers and Speakers - 99

**The Doctor's Armchair.**—Medical Sanity - 100

**Metropolitan National Nursing Association** - 101

**Shall the Radcliffe Infirmary be Rebuilt?** - 101

**Notes and News** - 102

**General Charities** - 103

**Scraps and Gleanings** - 103

**Annotations.**—Another Lupus Cure—Sixty Thousand Suicides—Small-pox and Cow-pox - 104

**For Hospital Sunday Readers, 1891.**—A Live-long Day - 105

EXTRA SUPPLEMENT—THE NURSING MIRROR.

**En Passant.**—Registration of Midwives—Nursing at the Homœopathic—A Special Performance—A Suburban Association—Short Items—Wirral Union - xlix

**Medical Petition Against Registration** - l

**Everybody's Opinion.**—The Need for Pension Funds—Pensions for Servants—St. John's Convalescent Home—District Nurses at Hartlepool—Pennies a Necessity - li

**Warwick Association** - li

**The Hospitals Association** - lii

**Appointments—Presentation** - lii

**Death in Our Ranks** - lii

**Wants and Workers** - lii

**Notes and Queries** - lii

**Amusements and Relaxation** - lii

